# T Cell Contamination in Flow Cytometry Gating Approaches for Analysis of Innate Lymphoid Cells

**DOI:** 10.1371/journal.pone.0094196

**Published:** 2014-04-23

**Authors:** Sara H. Burkhard, Florian Mair, Kathrin Nussbaum, Sabrina Hasler, Burkhard Becher

**Affiliations:** Institute of Experimental Immunology, University of Zurich, Zurich, Switzerland; University Medical Center of the Johannes Gutenberg University of Mainz, Germany

## Abstract

Innate lymphoid cells (ILCs) differ from T and B cells as they do not express genetically rearranged antigen receptors. The most prominent member of this group, NK cells, can be identified by numerous surface receptors such as natural cytotoxicity receptors (NCRs). However, novel groups of ILCs have recently been described and classified based on fate-determining transcription factors and cytokines being produced, similarly to T helper cells. Due to the lack of exclusive markers, ILCs are primarily defined by the paucity of lineage markers. Using *RORc*-fate-mapping mice, we found that the common lineage exclusion using CD3 yields an ILC population containing a large proportion of T cells with recombined TCR loci and low expression of CD3. Thus, we suggest adding CD5 as a marker for thorough elimination of T cells to avoid erroneous interpretations of ILC function in immunity.

## Introduction

The term innate lymphoid cells (ILCs) unifies a group of cells that are developmentally related and lack most lineage markers. They share morphological features with lymphocytes but, in contrast to B and T cells, they do not express recombined antigen receptors and can therefore be classified as innate immune cells [1]. In the past years, ILCs have been shown to be critical for physiological processes like lymphoid organ development [2], tissue homeostasis [3], early control of pathogens [4], but they can also promote inflammation if inadequately activated [5], [6]. Based on their cytokine and transcription factor expression profile ILCs have recently been given a uniform nomenclature, dividing ILCs in three main groups and thereby broadly reflecting the classification of T helper cell subsets [7]. The first subset (Group 1 ILCs) depends on expression of T-bet, leading to expression of IFN-γ [8], [9]. The second subset (Group 2 ILCs) expresses Gata3 and is characterized by the secretion of type 2 cytokines such as IL-13 and IL-5 [10], [11]. Group 3 ILCs (hereafter referred to as ILC3s) depend on the expression of the transcription factor retinoic acid-related orphan nuclear receptor Retinoid acid receptor-related orphan receptor (ROR)γt for their development as well as their function and secrete IL-22 and IL-17 [12], [13].

The most abundant ILC subtype are natural killer (NK) cells that can be characterized by expression of specific phenotypic markers, many of them belonging to the family of natural cytotoxicity receptors. However, for more recently discovered ILC subtypes such exclusive markers have yet to be defined. Therefore, during flow cytometric analysis of ILCs usually a combination of lineage markers is used to exclude other immune cells. As the phenotypic appearance of ILCs and their cytokine secretion pattern in many models resemble that of T cells, the elimination of T lymphocytes is absolutely critical to analyze phenotypic and functional properties of ILCs. Hence, many studies have been conducted using animals deficient for the recombination activating genes (*RAG*) 1 or 2, thereby genetically eliminating B and T cells [5], [8].

However, for some experimental models and studies on human tissues this is not an option, thus T cells have to be excluded by their expression of T cell specific markers. Using flow cytometry, separation of ILCs from T cells is most frequently achieved by staining for CD3, a part of the T cell receptor (TCR) complex. As T cells are known to modulate their CD3 expression level in particular following their activation [14] and usually outnumber the rare ILC subgroups, a clear discrimination between these two populations is essential.

Based on exemplary analysis of RORγt dependent ILC3s, we show here that exclusion of T cells using solely gating on CD3 leaves a significant contamination with T cells. These T cells were identified by staining for CD5, a surface marker that is involved in modulating TCR signaling. Depending on the organ analyzed, the fraction of T cells within the gated ILC3 population accounted for up to 80% of the remaining ILC3s, which in certain studies may falsify subsequent results. Quantitative PCR analysis for the constant regions of the T cell receptor α and δ chain (*TRAC* and *TRDC*) revealed that the remaining CD5^+^ cells belonged mainly to the αβ T cell lineage. In order to obtain a non-ambiguous separation of ILCs and T cells, stainings for different T cell surface molecules need to be combined. We therefore suggest complementing a CD3 and TCRβ staining with an antibody binding the CD5 molecule.

## Results and Discussion

### CD5^+^ fraction remains in the ILC3 gate after exclusion of T cells using CD3 and TCRβ

Besides its importance for ILC3s, RORγt is also involved in the development of T cells, mainly T_H_17 and γδ T cells [13], [15]. However, most ILC3 and T cell subsets will subsequently downregulate the expression of RORγt [16]. By breeding a transgenic mouse expressing the Cre recombinase under the control of the *RORc* promoter (driving RORγt) to a *ROSA26*-stop^fl/fl^ eYFP reporter mouse yielding in *RORc*-eYFP mice, the fate of these YFP positive RORγt -dependent cells can be mapped [17].

**Figure 1:**
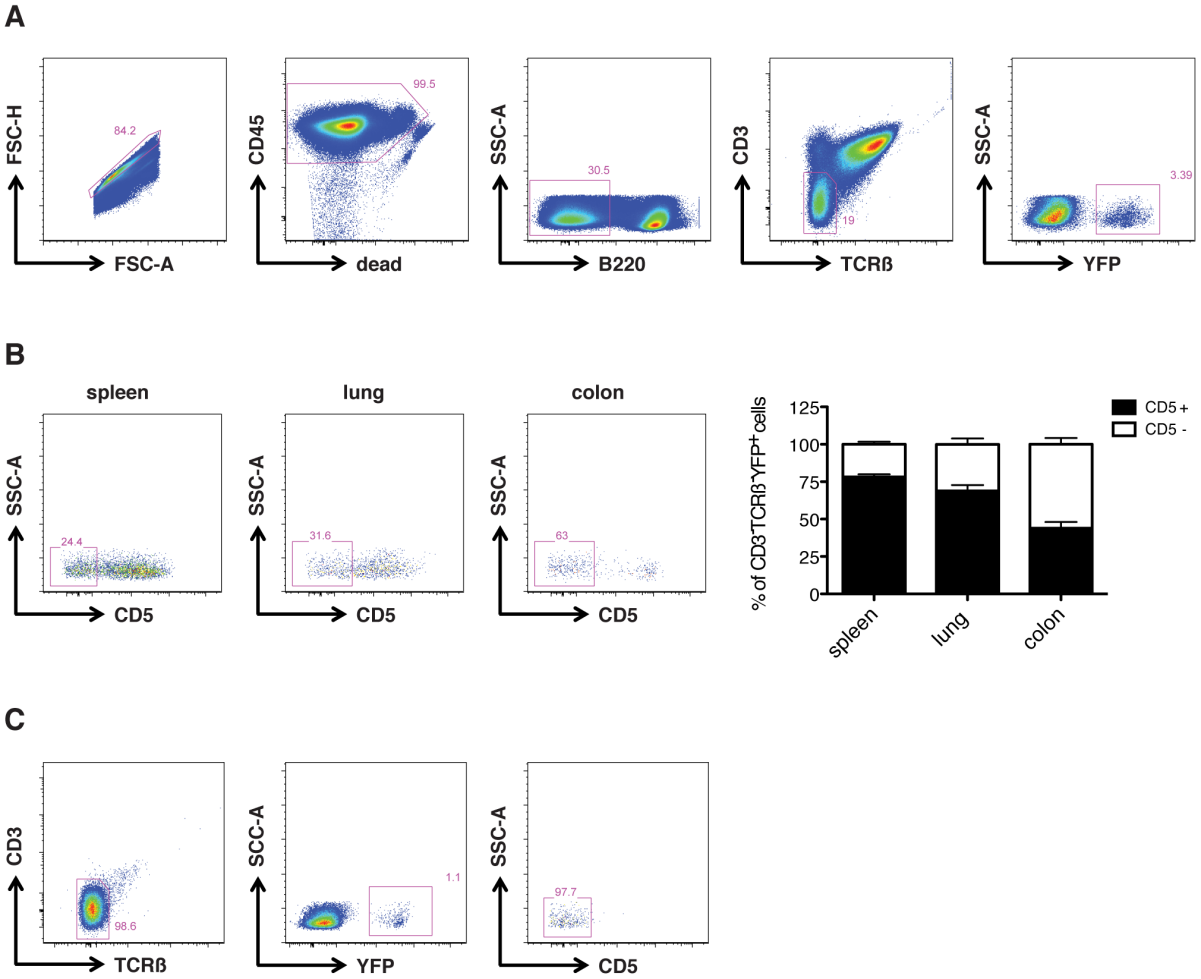
Flow cytometric analysis of ILC3s using *RORc*-eYFP fatemap mice. (a) Gating strategy for ILC3s in *RORc*-eYFP mice: after exclusion of doublets, live CD45^+^ B220^−^ CD3^−^ TCRβ^−^ YFP^+^ cells were selected. (b) The population gated in panel a contains a significant fraction of CD5^+^ cells both in spleen, lung and colon. (c) In *RORc*-eYFP-RAG^−/−^ mice all YFP^+^ CD45^+^ live cells stain negative for CD3, TCRβ and also CD5. Representative plots for n = 3 (mean +/− s.e.m.).

Using leukocytes isolated both from lymphoid (spleen) and non-lymphoid organs (colon and lung), we compared different gating approaches for the efficiency in separating ILC3s and T cells. The gating strategy involved exclusion of doublets, dead cells and B220 expressing events from CD45^+^ leukocytes. In order to exclude T cells, CD3 and TCRβ positive events were negatively selected followed by gating on YFP expressing cells (Figure 1a). Similar gating approaches are used to characterize both murine and human ILCs but are often not depicted in any figure or involve gating out multiple lineage markers in one fluorescent channel [10], [18]–[20]. However, upon further scrutiny of the assumed ILC3s, we discovered a significant percentage of these cells to stain positive for CD5, a molecule described to inhibit the T and B cell receptor signaling and was historically used as a pan T cell marker. Whereas in the colon this frequency was relatively low with 43.9% CD5^+^ cells within the ILC3 gate, in spleen and lung it reached 78.2% and 68.9%, respectively (Figure 1b). Strikingly, in *RORc*-eYFP fate mapping mice deficient for *RAG1* no expression of CD5 was detected, suggesting that the CD5^+^ cells obtained from non-RAG mice belong to the T cell lineage (Figure 1c). Notably, as CD5 is upregulated upon TCR engagement, it is highly expressed in activated TCR^low^ and CD3^low^ T cells, which will appear close to the ILC gate [21].

### CD5^+^ cells remaining within the CD3^−^ TCRβ^−^ ILC3 gate consist of αβ T cells

**Figure 2:**
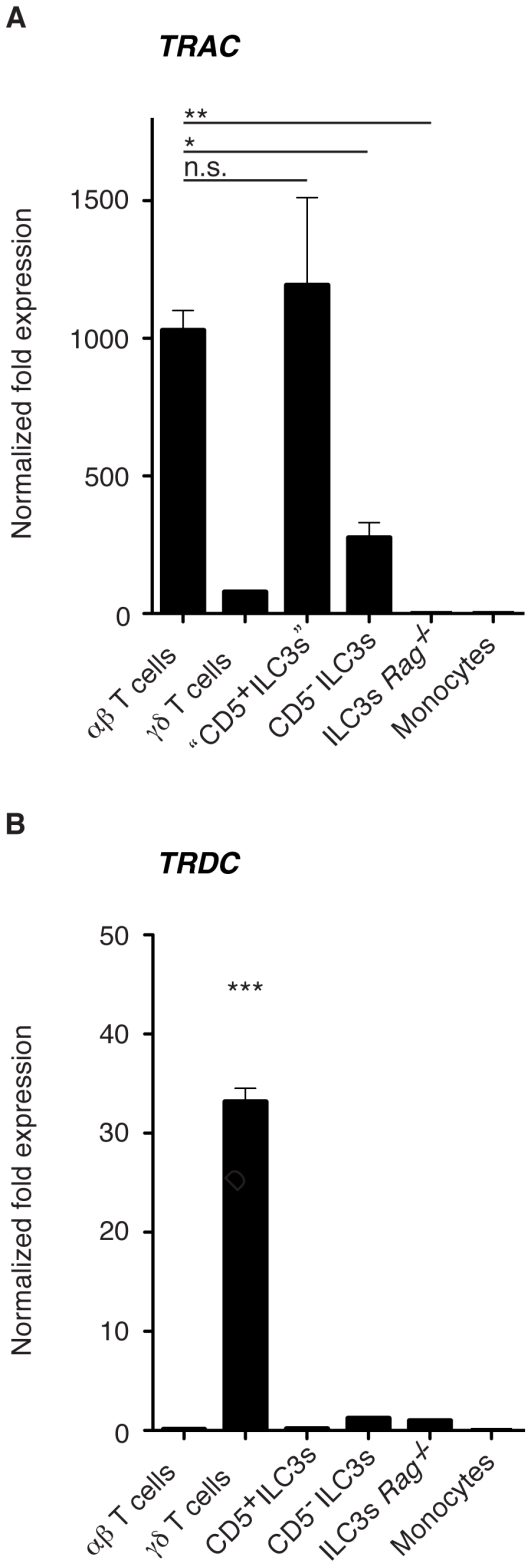
CD5^+^ positive cells within the ILC3 gate express high levels of *TRAC*. Normalized fold expression for *TRAC* and *TRDC* comparing CD5^+^ and CD5^−^ cells within the ILC3 gate (“CD5^+^ ILC3s”, pregated on CD45^+^ B220^−^ CD11b^−^ CD3^−^ TCRβ^−^ YFP^+^ cells), αβ and γδ T cells, monocytes (Mono) purified from *RORc*-eYFP mice and ILC3s derived from *RORc*-eYFP *RAG1* ^−/−^ mice (ILC3s *RAG1* ^−/−^). Results are presented relative to those obtained from ILC3s of *RORc*-eYFP *RAG1* ^−/−^ mice (mean +/− s.e.m.).

To determine the nature of the CD5 expressing CD3^−^ TCRβ^−^ cells, this population was purified by flow cytometric cell sorting from the spleen (Figure 1a and b). The mRNA levels for the constant region of the T cell receptor α and δ chain (*TRAC* and *TRDC*) were compared to that of CD5^−^ ILC3s, αβ and γδ T cells as well as monocytes. As a genetic control we used ILC3s derived from *RORc*-eYFP *RAG1* ^−/−^ mice. Whereas CD3^−^ TCRβ^−^ CD5^−^ ILC3s showed minor expression of both *TRAC* and *TRDC* (probably reflecting inevitable contamination from the sort purification), purified αβ and γδ T cell populations as expected transcribed high levels of *TRAC* or *TRDC*, respectively (Figure 2). Strikingly, when analyzing *TRAC* and *TRDC* expression levels in CD3^−^ TCRβ^−^ CD5^+^ cells we detected high levels of *TRAC*, suggesting that this population indeed consisted mainly of αβ T cells.

## Conclusions

We conclude that gating on lineage negative cells only using CD3 is not sufficient to firmly exclude αβ T cells from further analysis. This problem becomes particularly evident, when a CD3 containing “dump channel” is employed, where cells expressing lineage markers at different intensities are gated out in a single fluorescence channel. Furthermore, the scarcity of ILCs together with the high number of T cells, which depending on their activation status might express different levels of CD3, seems to cause limits for a separation based on one T cell marker. Thus, results obtained from contaminated ILC populations will be biased and T cells will contribute to the detected cytokine levels. This may not only produce artifacts but also mislead the characterization of ILC subsets according to the nomenclature based on cytokine and transcription factor expression.

Given that within the past years a strong association between ILC and T cell effector function has been proposed, we suggest that a thorough separation between these two populations is essential. Hence, lineage negative gating should not only be performed using CD3 only, but also CD5, preferably in a separate fluorescent channel.

## Materials and Methods

### Mouse strains

*RORc*-CRE and ROSA-stop^fl/fl^-eYFP mice, provided by Andreas Diefenbach, were bred to obtain *RORc*-eYFP fate mapping animals. Both strains were crossed to *RAG1* ^−/−^ mice, purchased from the Jackson Laboratory. Animals were between the ages of 6 to 16 weeks and kept under specific pathogen free conditions. All animal experiments were approved by the Swiss Cantonal Veterinary Office (license 147/2012, Zurich, Switzerland).

### Cell isolation

Mice were euthanized using CO_2_ inhalation and perfused with 40 ml cold PBS. Spleen, colon and lung were excised and processed as described below. **Spleens** were homogenized by mechanical disruption and filtered through a 70 µm mesh, followed by lysis of erythrocytes. **Lungs** were cut into small pieces, followed by 60 min of digestion at 37°C with 1 mg/ml Collagenase D (Roche) and 0.5 mg/ml DNAse (Sigma) in IMDM containing 25 mM HEPES and 2% fetal calve serum (FCS). Remaining pieces of tissue were homogenized using syringes and 18 gauge needles, followed by filtration through a 70 µm mesh. **Colons** were separated form the mesenteric fat. Luminal mucus was removed mechanically followed by incubation in Ca^2+^ Mg^2+^ lacking HBSS containing 2% of FCS, 1 mM DTT and 1.35 mM EDTA for 15 min at 37°C. After further incubation in HBSS complemented with EDTA for 30 min at 37°C the colons were cut and digested using 0.4 mg/ml collagenase IV (Sigma Aldrich) for 45 min at 37°C. The samples were then homogenized using a syringe with an 18 gauge needle and filtered through a 70 µm cell strainer.

### Flow cytometry

All flourochrome-conjugated antibodies used were obtained either from BD, BioLegend or eBioscience, and stainings were performed according to standard procedures for 20 minutes at 4°C. In all stainings, dead cells were excluded using an Aqua Live/Dead fixable staining reagent (Invitrogen), and doublets were excluded by FSC-A vs FSC-H gating. Analysis was performed using a LSR II Fortessa (special order research product, BD) with four laser lines (405 nm, 488 nm, 561 nm and 640 nm). Cell sorting experiments were carried out using a FACSAria III (BD). Data analysis was done using FlowJo V9.x (Treestar).

### mRNA extraction and quantitative real time PCR

Purified cells were resuspended in Trizol (Invitrogen) and kept at -80°C. RNA was extracted using the PureLink RNA Micro Kit (Invitrogen) according to the manufactures. RNA concentration was determined using a spectrophotometer (NanoDrop). Subsequent cDNA synthesis was performed using Superscript II rerverse transcriptase (Invitrogen) and oligo (dt) primers (PeproTech). Quantitative analysis was conducted using a SYBR Green master mix (Roche), *TRAC* and *TRDC* specific primers (see below) and measured by the C1000 Touch thermal cycler (BioRad). mRNA levels were determined by the cycle threshold values and normalized to the expression of the *Pol2* gene. Primers were designed using CLC Workbench: *TRAC*: ACAAGCTTCACCTGCCAA forward, GCTTTTCTCAGTCAACGTGG reverse; *TRDC*: TAGTCTCCTCATGTCAGCCC reverse, CTACGACTGCTGTTTGCCA forward. Statistical significance was determined using the BioRad CFX Manager 3.0.
